# Child Well‐Being and Family Quality of Life During the COVID‐19 Pandemic

**DOI:** 10.1111/cch.70063

**Published:** 2025-03-14

**Authors:** Conné Lategan, Amanda S. Newton, Jennifer Thull‐Freedman, Jianling Xie, Kathleen Winston, Bruce Wright, Michael Stubbs, Stephen B. Freedman

**Affiliations:** ^1^ Faculty of Medicine & Dentistry University of Alberta Edmonton Alberta Canada; ^2^ Department of Pediatrics, Faculty of Medicine & Dentistry University of Alberta Edmonton Alberta Canada; ^3^ Department of Pediatrics and Emergency Medicine, Cumming School of Medicine University of Calgary Calgary Alberta Canada; ^4^ Department of Pediatrics, Cumming School of Medicine University of Calgary Calgary Alberta Canada; ^5^ Department of Psychiatry, Cumming School of Medicine University of Calgary Calgary Alberta Canada; ^6^ Departments of Pediatrics and Emergency Medicine, Cumming School of Medicine University of Calgary Calgary Alberta Canada

**Keywords:** child, COVID‐19, emergencies, mental health, pandemics, quality of life, SARS‐CoV‐2, well‐being

## Abstract

**Background:**

Little is known about changes in child well‐being and family quality of life (QoL) among children seeking emergency department care because of mental health concerns over the course of the pandemic.

**Methods:**

Prospective cohort study of children < 18 who visited two paediatric EDs in Alberta, Canada, for an acute mental health concern. Early and late pandemic time periods were defined as 15 March 2020–14 March 2021 and 1 July 2021–30 June 2022, respectively. The Stirling Children's Well‐being and Warwick–Edinburgh Mental Well‐being scales quantified well‐being; the Family Quality of Life Scale assessed family QoL. These scales were completed as soon as possible following the ED visit. Linear regression models assessed the association between pandemic period and the change in well‐being and family QoL.

**Results:**

One thousand four hundred four children were enrolled during the study time periods (50.4% early, 49.6% late). Seventy‐two percent (1009/1404) of participants were White, 53.8% (744/1404) were female, and the median age was 13 (IQR, 11–15) years. Well‐being remained unchanged between time periods, whereas family QoL was lower in the late pandemic time period than in the early period (mean difference: −2.16, 95% CI: −3.79, −0.53; *p* = 0.01). Among children < 13 years, previous mental health care and requiring inpatient admission were negatively associated with well‐being. Having an autism diagnosis and a comprehensive ED mental health evaluation were negatively associated with well‐being in older children.

**Conclusions:**

Reduced family QoL may signal stressors experienced by caregivers and the lingering consequences of the pandemic. Resources that address well‐being and support the family unit are needed to improve the mental health of children.

**Abbreviations**
COVID‐19: coronavirus
ED: emergency department
FQOL: Family Quality of Life Scale
LOS: length of stay
QoL: quality of life
SCWBS: Stirling Children's Well‐Being Scale
WEMWBS: Warwick–Edinburgh Mental Well‐being Scale

**Summary**

- What is known?
- Nearly 20% of children under the age of 18 years in the United States and Canada have a mental, emotional, developmental or behavioural disorder, and the number and severity of paediatric mental health emergency department visits increased during the pandemic, particularly during the early pandemic period.
- Little is known about the longer term impacts of the pandemic on child well‐being and family functioning.
- What is new?
- In this multicentre paediatric emergency department cohort study of 1404 children, children's well‐being scores remained unchanged, whereas family quality of life was reduced in the later pandemic time period.
- Having a history of mental health concerns, autism and requiring greater resources at the index visit were associated with lower well‐being scores.
- What is significant for clinical practice?
- Although overall child well‐being did not decline during the pandemic, family quality of life did.
- Measures to support the family unit through future pandemics should be an important public health consideration.

## 1 Introduction

Nearly 20% of children under the age of 18 years in the United States and Canada have a mental, emotional, developmental or behavioural disorder (2022 National Healthcare Quality and Disparities Report 2022; The Conference Board of Canada 2023). The proportion of paediatric emergency department (ED) visits for mental health concerns doubled during the 10 years preceding the coronavirus (COVID‐19) pandemic (Bommersbach et al. 2023). This trend continued during the pandemic with relative increases in visits for attempted suicide, self‐harm (Mitchell et al. 2023) and suicidal ideation (Madigan et al. 2023; Poonai et al. 2023). The increase in the frequency and severity of paediatric ED mental health visits during the pandemic has been attributed to disruptions to in‐person schooling and connectedness among peers (Tsujimoto et al. 2022), lack of access to school‐based mental health supports (Erjavac et al. 2023) and fewer opportunities for social interactions and extracurricular activities (LaForge‐MacKenzie et al. 2022; Neville et al. 2022; T. Vaillancourt et al. 2021). Other factors include increases in domestic violence (Letourneau et al. 2022), parental anxiety and stress (Meade 2021; Racine et al. 2021) and reduced family functioning (Wolf and Schmitz 2023).

The impact of the pandemic on the number and severity of paediatric mental health ED visits varied over time, with rates being highest during the first period of school closures (i.e., the early pandemic period; Newton et al. 2023). The increase was most marked for visits related to suicide and self‐injury and among those children with pre‐existing mental health diagnoses (Shankar et al. 2022). These findings may reflect the impact of public health prevention measures, anxiety over the loss of loved ones, economic uncertainty and disruptions to the normal school and extracurricular routines of children during the early phase of the pandemic (Chaabane et al. 2021; Singh et al. 2020; Viner et al. 2022). Little is still known about the longer term impacts of the pandemic on child well‐being and family functioning.

To advise on how best to support the well‐being of children and adolescents, a better understanding is needed of how well‐being was affected by the challenging circumstances of the pandemic (Holmes et al. 2020). As previous research has shown the importance of interventions that promote home connectedness, to support the mental health of children, an understanding of family functioning is important. In this study, we explored the mental well‐being of children and adolescents and quality of life (QoL) among their families during the COVID‐19 pandemic by comparing these between children who visited a paediatric ED for mental health concerns early in the pandemic to those who visited during the late phase of the COVID‐19 pandemic.

## 2 Methods

### 2.1 Study Design

Data were collected as part of an intervention implementation study designed to improve mental health care conducted between 29 January 2020 and 30 June 2022 in two paediatric EDs in Alberta, Canada (Freedman et al. 2020). The implementation study was designed to evaluate the integration of an acute care bundle into routine clinical care. The components include assessing self‐harm risk at triage using the Ask Suicide‐Screening Questionnaire to standardize the questions administered and enable risk stratification (Horowitz et al. 2013), use of the HEADS‐ED to support focused mental health evaluations (Cappelli et al. 2020) and implementation of a Choice And Partnership Approach to enable shared decision‐making in care following the ED visit (Clark et al. 2018).

Caregivers of eligible participants provided consent; assent was obtained when appropriate. Children ≥ 14 years of age who presented without a legal guardian participated as mature minors. Data were collected via email survey as soon as possible following the ED visit. Ethics approval was obtained from the institutional research ethics boards. Results are reported in accordance with the Strengthening the Reporting of Observational Studies in Epidemiology guidelines (von Elm et al. 2007).

### 2.2 Study Population

Enrolled children < 18 years old presented to the ED with any of the following chief complaints: anxiety, bizarre behaviour, concern for patient's welfare, depression, suicidality and nonsuicidal self‐injury, paediatric disruptive behaviour, insomnia (secondary to anxiety or worries), situational crisis or violent or homicidal behaviour. Children excluded from the study were those brought to the ED by a police/peace officer or ambulance, with a communication barrier at triage, or requiring medical evaluation and treatment before mental health care evaluation (e.g., hallucinations/delusions and syndromes associated with physiologic disturbances such as eating disorders and severe self‐injury). These exclusion criteria were required as children with these features could not receive the intervention in the implementation study as it altered the clinical care provided to children with acute mental health concerns (Freedman et al. 2020). We also excluded children who previously participated in the study to ensure independence of observations.

### 2.3 Study Periods

We used publicly available data on pandemic‐related initiatives in Alberta to identify early (15 March 2020–14 March 2021) and late (1 July 2021–30 June 2022) pandemic time periods. The early period reflects the first 12 months following the implementation of provincial public health restrictions, and the late period reflects the last 12 months before provincial public health restrictions were removed.

### 2.4 Outcome Measures

The primary outcome was child well‐being quantified using the Stirling Children's Well‐being Scale (SCWBS) for children aged < 13.0 years (Table S1) and the Warwick–Edinburgh Mental Well‐being Scale (WEMWBS) (Table S2) for those aged 13.0 to < 18.0 years. The SCWBS combines 12 questions across three domains (optimism, cheerfulness and relaxation; satisfying interpersonal relationships; clear thinking and competence) to provide an internally consistent, reliable and single‐dimensioned well‐being score (Liddle and Carter 2015). Scores range from 12 to 60 with higher scores representing a greater level of well‐being. The WEMWBS, which consists of 14 questions related to feelings and functioning, has excellent internal consistency and reliability (Bass et al. 2016; McKay and Andretta 2017; Tennant et al. 2007). Scores range from 14 to 70 with higher scores representing a greater level of well‐being.

The a priori planned secondary outcome was family QoL measured using the Family Quality of Life Scale (FQOL) (Table S3). The FQOL consists of 25 items spread across five subscales: family interaction (6 items; score range 6–30), parenting (6 items; score range 6–30), emotional well‐being (4 items; score range 4–20), physical and material well‐being (5 items; score range 5–25) and disability‐related support (4 items; score range 4–20) (Park et al. 2003; Rivard et al. 2017; Summers et al. 2005). Total scores range from 25 to 125 with higher scores corresponding to a higher family QoL (Summers et al. 2005). Well‐being and family QoL data were collected after the ED visit via telephone or an online questionnaire completed by the parents/caregivers and/or the child. Each participating child/parent dyad only provided one set of scores.

### 2.5 Data Collection

Initial contact with all families was made by a member of the circle of care who requested permission for a member of our research team to contact them. If consent to be contacted was provided, then a research assistant contacted the family and obtained informed consent and assent as appropriate. All telephone calls occurred as soon as possible following the ED visit to minimize the time interval between the ED visit and data collection.

Baseline demographic information (age, gender identity, ethnoracial background), history of neurodevelopmental conditions (autism spectrum disorder, developmental delay) and mental health care (current receipt of outpatient mental health care, prior hospitalizations and/or ED visits for mental health concerns) were collected as soon as possible after the ED visit via telephone or an online questionnaire completed by the parents/caregivers and/or the child. Telephone‐based data collection was conducted in a standardized manner by trained research assistants. Online data collection occurred through the study's REDCap database.

The rationale for including race and ethnicity in the analysis relates to the associations between these variables and identified disparities in the recognition and treatment of mental health disorders in Canadian and US children (Georgiades et al. 2018; Kamali et al. 2023; Saunders et al. 2018). Race and ethnicity were reported by caregivers.

ED visit data (discharge diagnosis, length of stay [LOS], psychiatry consultation, comprehensive mental health evaluation, disposition, return ED visits for mental health care) were extracted from the electronic medical record by trained research assistants. Discharge diagnoses were coded during extraction using the International Classification of Diseases, Version 10 (ICD‐10) F codes for mental and behavioural disorders and R and X codes for intentional self‐harm.

### 2.6 Data Analysis

We reported demographic data of participants based on study period using counts and percentages for categorical variables and median and IQR for continuous variables. The SCWBS and WEMWBS total scores were calculated by summing the individual 12‐ and 14‐item well‐being items, respectively. Total and subscale FQOL scores were also calculated. Unadjusted comparisons of SCWBS, WEMWBS and FQOL total scores and subscale FQOL scores between the early and late pandemic time periods were conducted with *t*‐tests. These are not repeat measures for a parent/child dyad; rather, each participating parent/child dyad were enrolled in one period only (i.e., early or late), and their scores were thus included in only one of these periods.

Linear regression analyses with backwards elimination were used to evaluate the adjusted association between pandemic time period and child well‐being and FQOL scores. A *p*‐value threshold of 0.05 was used for retention in the final reduced model for the following independent variables: patient demographics (age, gender identity, ethnoracial background) and health history (history of autism spectrum disorder or developmental delay, receiving outpatient mental health care at the time of the ED visit, prior hospitalization or ED visit related to mental health) and ED visit characteristics (discharge diagnosis, LOS, comprehensive mental health evaluation, psychiatry consultation, hospital admission or transfer to inpatient care). Although some ethnoracial background categories were combined in the regression analysis due to small cell sizes. Analyses were conducted using SPSS 25.0 (Armonk, NY: IBM Corp.). Statistical tests were two‐tailed and *p*‐values of < 0.05 were considered statistically significant.

## 3 Results

### 3.1 Study Participants

**FIGURE 1:**
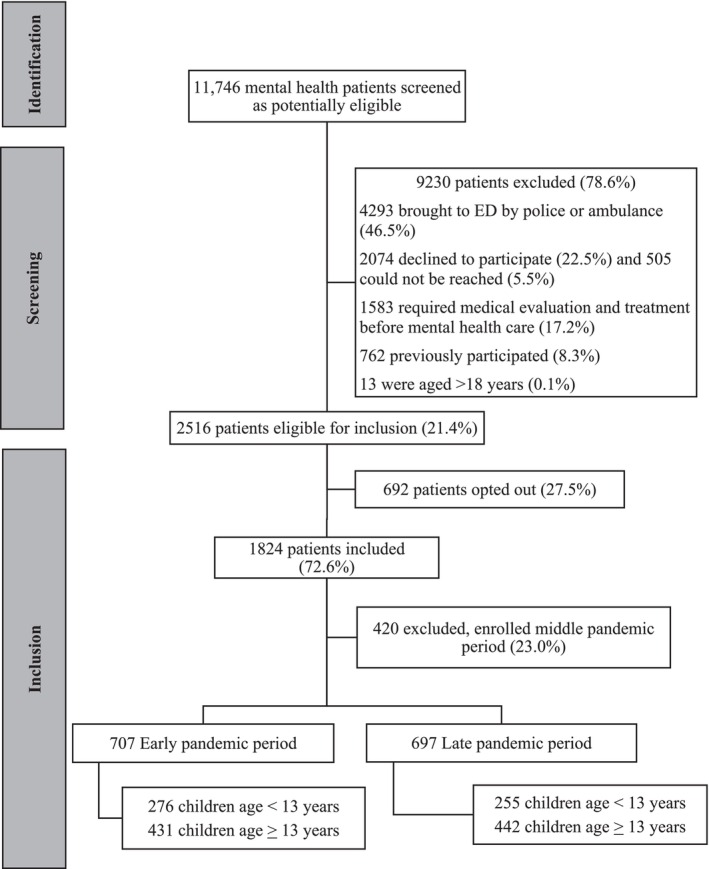
Flow of participants through the study.

The cohort included 1404 children of the 1824 that were enrolled in the implementation study (76.9%) as 420 were enrolled during the implementation period and thus did not meet our study definition of early or late periods (Figure 1). Participant median age was 13 (IQR: 11, 15) years, 71.9% (1009/1404) were White, and 53.0% (744/1404) identified as being female. Demographic characteristics were similar across the two time periods except that those enrolled during the early pandemic period had more prior ED visits and hospitalizations related to mental health concerns (Table 1). The most common discharge diagnosis during both time periods was suicidal ideation (37.7% early vs. 38.8% late; difference: 1.1% [95% CI: −4.1%, 0.63%]). During the early pandemic period, greater proportions of children had a psychiatric consultation (25.4% early vs. 18.6% late; difference: −6.8% [95% CI: −11.2%, −2.4%]) and were admitted to hospital (14.2% early vs. 9.1% late; difference: −5.1% [95% CI: −8.5%, −1.7%]). Children presenting to the ED during the early pandemic period had a shorter median LOS (4.2 h [IQR 2.8, 6.7] and 5.8 h [IQR 3.6, 8.7]; difference: 1.1 h [95% CI: 0.8, 1.5]) and a lower proportion left the ED without being seen by a physician (1.6% early vs. 6.3% late; difference: 4.7% [95% CI: 2.6%, 6.7%]; Table 2).

**TABLE 1:** Participant characteristics—Overall and stratified by pandemic study period.

	Total (*n* = 1404)	Early pandemic period (*n* = 707)	Late pandemic period (*n* = 697)	*p*
Age in years, median IQR	13 (11, 15)	13 (11, 15)	14 (11, 15)	0.16
Age group, *n* (%)				0.59
≤ 5 years	18 (1.3)	11 (1.6)	7 (1.0)	
6–12 years	513 (36.5)	265 (37.5)	248 (35.6)	
13–17 years	873 (62.2)	431 (61.0)	442 (63.4)	
Ethnoracial background, *n* (%)				0.76
White	1009 (71.9)	498 (70.4)	511 (73.3)	
Multiple groups^a^	113 (8.0)	63 (8.9)	50 (7.2)	
First Nations, Inuit or Métis	101 (7.2)	50 (7.1)	51 (7.3)	
South, Southcentral or Southeast Asian	86 (6.1)	47 (6.6)	39 (5.6)	
Indeterminate	36 (2.6)	17 (2.4)	19 (2.7)	
Black	28 (2.0)	15 (2.1)	13 (1.9)	
Latin American	28 (2.0)	14 (2.0)	14 (2.0)	
Declined to answer or missing	2 (0.1)	2 (0.3)	0 (0)	
West Asian	1 (0.1)	1 (0.1)	0 (0)	
Gender identity, *n* (%)				0.12
Female	744 (53.0)	387 (54.7)	357 (51.2)	
Male	517 (36.8)	263 (37.2)	254 (36.4)	
Non‐binary	73 (5.2)	28 (4.0)	45 (6.5)	
TransMale	31 (2.5)	11 (1.6)	20 (2.9)	
TransFemale	4 (0.3)	2 (0.3)	2 (0.3)	
Declined to answer or missing	35 (2.5)	16 (2.3)	19 (2.7)	
History of autism spectrum disorder, *n* (%)^b^	88 (6.4)	53 (7.6)	35 (5.1)	0.05
History of developmental delay, *n* (%)^b^	184 (13.3)	94 (13.5)	90 (13.1)	0.82
Currently receiving outpatient mental health care, *n* (%)^b^	1140 (82.5)	577 (83.0)	563 (82.0)	0.60
Prior hospitalization related to mental health, *n* (%)^c^	199 (14.4)	124 (17.9)	75 (10.9)	< 0.001
Prior ED visit related to mental health, *n* (%)^c^	383 (27.7)	230 (33.1)	153 (22.3)	< 0.001

^a^ Participants who selected more than one ethnoracial background category.

^b^ Based on 1382/1404 (98.4%) responses.

^c^ Based on 1381/1404 (98.4%) responses.

**TABLE 2:** Emergency department visit characteristics—Overall and stratified by pandemic study period.

	Total *N* = 1350^a^	Early pandemic period *N* = 682	Late pandemic period *N* = 668	*p*
Discharge diagnosis, *n* (%)^a^				
Suicidal ideation	516 (38.2)	257 (37.7)	259 (38.8)	0.68
Neurotic, stress‐related or somatoform disorder	331 (24.5)	186 (27.3)	145 (21.7)	0.02
Mood disorder	325 (24.1)	171 (25.1	154 (23.1)	0.39
Other diagnosis related to mental health needs	281 (20.8)	123 (18.0)	158 (23.7)	0.01
Behavioural or emotional disorder	110 (8.1)	74 (10.9)	36 (5.4)	< 0.001
Intentional self‐harm not requiring medical care	66 (4.9)	27 (4.0)	39 (5.8)	0.11
Disorder of personality or behaviour	21 (1.6)	12 (1.8)	9 (1.3)	0.66
Disorder of psychological development	20 (1.5)	14 (2.1)	6 (0.9)	0.11
Mental/behavioural disorder due to substance use	3 (0.2)	1 (0.1)	2 (0.3)	0.62
Unspecified mental disorder	1 (0.1)	1 (0.1)	0 (0)	> 0.99
Missing/not documented	81 (6.0)	24 (3.5)	57 (8.5)	< 0.001
Length of stay, hours, median IQR	4.9 (3.0, 8.0)	4.2 (2.8, 6.7)	5.8 (3.6, 8.7)	< 0.001
Comprehensive mental health evaluation, *n* (%)^b^	891 (66.0)	421 (61.7)	470 (70.5)	< 0.001
Psychiatry consultation, *n* (%)^b^	297 (22.0)	173 (25.4)	124 (18.6)	0.003
Disposition, *n* (%)				
Hospital admission or transfer to inpatient care^b^	158 (11.7)	97 (14.2)	61 (9.1)	0.004
Left without being seen	53 (3.9)	11 (1.6)	42 (6.3)	< 0.001
Return ED visit for mental health care^b^				
Within 72 h	24 (1.8)	8 (1.2)	16 (2.4)	0.10
Within 30 days	128 (9.5)	58 (8.5)	70 (10.5)	0.21

^a^ Based on 1350/1404 (96.2%) consented to ED chart review.

^b^ Based on 1349/1350 (99.9%) consented to ED chart review.

### 3.2 Child Well‐Being and Family QoL

**FIGURE 2:**
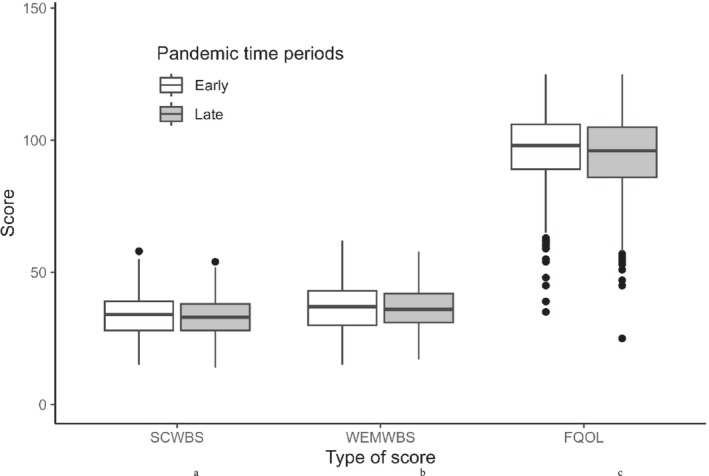
Box plots of the well‐being and the family quality of life scores by pandemic time phases. FQOL: Family Quality of Life Scale (range: 25–125); SCWBS: Stirling Children's Wellbeing Scale score (range:12–60); WEMWBS: Warwick–Edinburgh Mental Wellbeing Scale score (range: 14–70). ^a^SCWBS mean difference: −0.43 (95% CI: −1.61, 0.76); *p* = 0.48. ^b^WEMWBS mean difference: −0.53 (95% CI: −1.87, 0.83); *p* = 0.45. ^c^FQOL mean difference: −2.16 (95% CI: −3.79, −0.53); *p* = 0.01.

Well‐being and FQOL data were collected at a median of 14.7 (9.7, 23.2) and 12.1 (10.1, 17.7) days after the index visit during the early and late pandemic periods, respectively. Mean well‐being scores for children did not differ between the early pandemic period and the late pandemic period (difference: −0.43 [95% CI: −1.61, 0.76] for children < 13 years, and −0.53 [95% CI: −1.87, 0.83] for children ≥ 13 years; Table S4 and Figure 2). The item ‘I think lots of people care about me’ received the highest mean score among children < 13 years in both pandemic periods (early period mean: 3.18 ± 1.13 vs. late period mean: 3.08 ± 1.08; Table S5). The items with the lowest mean score were ‘I've been feeling relaxed’ (mean: 2.45 ± 0.86) and ‘I've been feeling calm’ (mean: 2.44 ± 0.86) in the early and late pandemic periods, respectively. Among children ≥ 13 years, the item ‘I've been feeling loved’ received the highest mean score in both pandemic periods (early period mean: 3.27 ± 0.96 vs. late period mean: 3.29 ± 0.89; Table S6). The item with the lowest mean score in the early pandemic period was ‘I've had energy to spare’ (mean: 2.31 ± 0.94), whereas the items ‘I've been feeling good about myself’ (mean: 2.32 ± 0.85) and ‘I've been feeling confident’ (mean: 2.32 ± 0.84) received the lowest mean score in the late pandemic period.

Mean FQOL scores were lower during the late pandemic period (mean difference: −2.16 [95% CI: −3.79, −0.53]; *p* = 0.01). Three out of the five FQOL subscales were lower during the late pandemic period: family interaction (mean difference: −0.55 [95% CI: −1.04, −0.06]; *p* = 0.03), parenting (mean difference: −0.60 [95% CI: −1.03, −0.17]; *p* = 0.007) and physical/material well‐being (mean difference: −0.56 [95% CI: −0.92, −0.21]; *p* = 0.002; Table S7).

### 3.3 Associations With Well‐Being and Family QoL

Self‐identifying as having an Asian ethnoracial background and those who did not disclose or had an unknown ethnoracial background was associated with higher well‐being scores in children < 13 years while receiving outpatient mental health care at the time of the ED visit, being admitted or transferred for inpatient care, and increasing age was negatively associated with well‐being (Table 3). Among children ≥ 13 years of age, self‐identifying Asian ethnoracial background and having a discharge diagnosis related to disorders of personality and behaviour were associated with higher well‐being scores while having a history of autism spectrum disorder and receiving a comprehensive evaluation by a mental health team member were negatively associated with well‐being scores (Table 3). Child well‐being was not associated with the pandemic time period in either age group.

**TABLE 3:** Regression analysis evaluating the adjusted association between the early and late pandemic time periods and well‐being scores.

Wellbeing scores among children < 13.0 years		
	Adjusted mean difference (95% CI)	*p*
Age, per year older	−0.5 (−0.77, −0.24)	< 0.001
Ethnoracial background		
Black, Latin American, West Asian	1.30 (−1.56, 4.16)	0.37
First Nations, Inuit or Métis	−0.97 (−3.31, 1.36)	0.42
Declined to answer or missing	8.37 (3.23, 13.52)	0.001
Multiple groups	0.60 (−1.40, 2.59)	0.56
South, Southcentral or Southeast Asian	3.80 (1.18, 6.42)	0.004
White	Reference	
Currently receiving outpatient mental health care		
Yes	−2.09 (−3.59, −0.6)	0.006
No	Reference	
Discharge diagnosis		
Suicidal ideation	−1.88 (−3.1, −0.66)	0.003
No diagnosis of suicidal ideation	Reference	
Hospital admission or transfer to inpatient care		
Yes	−3.27 (−5.21, −1.34)	< 0.001
No	Reference	
Pandemic time period		
Late	−0.73 (−1.88, 0.43)	0.22
Early	Reference	
Wellbeing scores among children 13.0–17.99 years		
Ethnoracial background		
Black, Latin American or West Asian	0.76 (−2.67, 4.20)	0.66
First Nations, Inuit or Metis	0.12 (−2.58, 2.83)	0.93
Declined to answer or missing	0.54 (−6.84, 7.92)	0.89
Multiple	−0.23 (−2.91, 2.45)	0.87
South, Southcentral or Southeast Asian	3.49 (0.89, 6.09)	0.009
White	Reference	
History of autism spectrum disorder		
Yes	−4.19 (−7.27, −1.11)	0.008
No	Reference	
Prior ED visit related to mental health		
Yes	−1.52 (−2.98, −0.07)	0.04
No	Reference	
Comprehensive mental health evaluation		
Yes	−1.44 (−2.94, 0.05)	0.06
No	Reference	
Discharge diagnosis		
Disorder of personality or behaviour	6.44 (1.21, 11.68)	0.02
No diagnosis of disorder of personality or behaviour	Reference	
Other diagnosis related to mental health needs	2.21 (0.38, 4.04)	0.02
No other diagnosis related to mental health needs	Reference	
Pandemic time period		
Late	−0.64 (−2.01, 0.74)	0.36
Early	Reference	

Abbreviation: ED, emergency department.

Family QoL was associated with having a discharge diagnosis related to mood disorders (adjusted mean difference: 3.6 [95% CI: 1.7, 5.5]) and currently receiving outpatient mental health care at the time of the ED visit (adjusted mean difference: 2.3 [95% CI: 0.1, 4.5]; Table S8). Characteristics that were negatively associated with family QoL included having a history of developmental delay, prior hospitalization for a mental health concern, receiving a psychiatry consultation and presenting to the ED during the late pandemic period.

## 4 Discussion

Among children with mental health crises who visited a paediatric ED during the COVID‐19 pandemic, well‐being did not differ between early and late pandemic periods. Conversely, the parents/caregivers of these children reported that family QoL decreased during the late pandemic period with the most notable QoL reductions for family interaction, parenting and physical/material well‐being.

Our findings align with the existing literature that describes how the impact of the pandemic varied over time. Others have reported that global mental health problems did not change between the first and second waves of the COVID‐19 pandemic; they did report a decrease in health‐related QoL (Ravens‐Sieberer et al. 2023). Emotional difficulties, peer‐related mental health challenges, anxiety, depression and psychosomatic symptoms also increased between the first and second pandemic waves (Ravens‐Sieberer et al. 2023). Research on the impacts of the third pandemic wave includes report of reductions in these symptoms relative to the second wave, but a prevalence that still exceeded pre‐pandemic levels (Ravens‐Sieberer et al. 2022). In a study of the fourth pandemic wave, impacts on parents persisted throughout the pandemic and they did not recover in a manner akin to what was seen in children (Ehrler et al. 2023). Our finding that family QoL was lower in the late pandemic time period than in the early time period extends these findings (Ehrler et al. 2023). The pandemic was an unforeseen and unpredictable period for parents and caregivers who may have experienced sudden increases in daily stressors related to financial security, job loss or underemployment, COVID‐19 infections or other illnesses, as well as changes in social supports and childcare (McGill et al. 2022; Prime et al. 2020). For the parents and caregivers in our study, they may have also assumed the responsibility of meeting the social and educational needs of their children when school and daycare closures occurred throughout the province (Prime et al. 2020). The lower ratings for the parenting and family interaction subscales in the late pandemic time period may reflect high levels of burnout, stress, familial strain and economic pressures (Adams et al. 2021; McGoron et al. 2022; Shahid et al. 2023), with a diminished emotional capacity among parents/caregivers to nurture and support family relationships (Prime et al. 2020; Richard et al. 2023). The decline in physical and material well‐being subscale scores for QoL may reflect the financial hardships and disruptions to the global supply chain and economy (Andrade et al. 2022).

In this study, we found that although receiving outpatient mental health care at the time of the ED visit was negatively associated with child well‐being, it was associated with higher family QoL scores. Although children with pre‐existing mental health conditions experienced deteriorations or exacerbations during the pandemic (Cost et al. 2022; Hawke et al. 2020; Richard et al. 2023; T. Vaillancourt et al. 2021; Theberath et al. 2022), there is evidence that a child's mental health condition can negatively impact parental well‐being (Azman et al. 2019; Mendenhall 2011). Our results may reflect how the existence of ongoing outpatient mental health treatment promotes the development of coping skills and family resilience (Setiawan et al. 2022). That parent–family connectedness is a protective factor for child mental health (Butler et al. 2022; Horowitz et al. 2020; Horwitz et al. 2021; Hua et al. 2024; Montero‐Marin et al. 2023) and family stress and instability are risk factors for poor child mental health suggests that family QoL is an important aspect to address during mental health care to identify resources that may be beneficial to families.

Health care factors identified as negatively associated with child well‐being—prior receipt of outpatient mental health care, prior ED visit for mental health concerns and requiring admission for inpatient care—raise important questions for how care is delivered and experienced by children. Previous work examining parental and child satisfaction with ED mental health care reported the highest rates of dissatisfaction are related to inadequate availability of resources for acute stabilization in the ED, the inability to be assessed by a particular health care professional (e.g., mental health team member or psychiatrist) during the ED visit and the fact that the child's symptoms were not eliminated (Lategan et al. 2023). Given the complex and longstanding nature of some mental health disorders in children which require resource‐intensive and continued care, ED visits may not adequately improve child mental health and well‐being and ongoing care beyond the ED is usually required (Ali et al. 2012; Reid et al. 2011). Future quality improvement initiatives should measure the impact that improved care has on child well‐being.

On a final note, self‐identifying as Asian was associated with higher well‐being scores. Although there is a paucity of Canadian literature examining the role ethnoracial background plays in child mental health, certain racialized groups including South Asian, Chinese, Filipino and Black ethnicities have reported lower rates of mood, anxiety and substance use disorders (Canada 2023a). In contrast, a study in the United States found that Black and Asian American adolescent children experienced significant challenges and worsening mental health symptoms during the pandemic (Eboigbe et al. 2023). However, protective factors were also identified, including strong familial and community support, ethnoracial socialization and identity. Although our study did not collect data on socio‐economic status, growing evidence connects economic inequality and poor mental health (Pickett and Wilkinson 2010). In fact, individuals with low socio‐economic status have higher prevalence rates of mental disorders than those with higher SES but are less likely to receive treatment (Niemeyer and Knaevelsrud 2023). However, as Canada's Asian population achieves above‐average educational degrees and representation in professional occupations (Canada 2023b), our finding may reflect these protective factors. Alternatively, it may be that Asian children were brought to the ED at a higher level of well‐being compared with children of other ethnoracial backgrounds.

This study has limitations. The results may be subject to non‐response and volunteer biases and thus possibly underrepresent important perspectives. Limiting survey completion to those without significant language barriers resulted in the omission of non‐English speaking perspectives. Further, we lacked representation of children in foster care or group homes in which legal guardian consent could not be obtained. Study design limitations prevented documentation of reasons of non‐participation and the inclusion of children with certain mental health presentations like schizophrenia or intentional self‐harm requiring medical intervention (Freedman et al. 2020). We also limited the participation of individuals to a single time point, that is, children presenting to the ED within the same or during both pandemic time periods, could only be enrolled once. Thus, we cannot conclude that the family QoL of individual children had declined over the course of the pandemic, limiting our interpretation to a population‐level understanding. Ideally, an evaluation of well‐being prior to, during and after the pandemic would have been conducted; however, our data were collected as part of a longitudinal study that was planned and launched prior to the pandemic, and the data collected and time periods could not be altered to overcome this limitation. Additionally, our study did not collect data on how hospital resources such as psychiatric bed availability fluctuated during the early and late pandemic periods. Reduced inpatient care capacity is a key consideration that could affect the well‐being of patients who may have benefited from hospitalization but were discharged because of a lack of bed availability. Future studies should consider bed availability when evaluating for the well‐being of children experiencing acute mental health crises. Lastly, family QoL may have been lower during the later pandemic period because of the removal of many social and financial supports that had been implemented during the early pandemic period, yet the pandemic and its effects were still ongoing.

## 5 Conclusion

In conclusion, although we found no difference in the well‐being of children seeking ED care for mental health concerns across the course of the pandemic, family QoL was lower during the last year of the COVID‐19 pandemic. Individual characteristics associated with child well‐being may reflect the severity threshold for seeking ED mental health care. Improved access to community‐based mental health is needed for those groups of children and families at increased risk for reduced well‐being and QoL.

## Author Contributions

**Conné Lategan:** conceptualization, data curation, investigation, methodology, writing – original draft. **Amanda S. Newton:** conceptualization, funding acquisition, investigation, methodology, project administration, supervision, writing – review and editing. **Jennifer Thull‐Freedman:** conceptualization, funding acquisition, investigation, methodology, project administration, writing – review and editing. **Jianling Xie:** formal analysis, investigation, methodology, software, writing – review and editing. **Kathleen Winston:** data curation, investigation, project administration, writing – review and editing. **Bruce Wright:** investigation, project administration, writing – review and editing. **Michael Stubbs:** investigation, project administration, writing – review and editing. **Stephen B. Freedman:** conceptualization, methodology, funding acquisition, investigation, project administration, resources, software, supervision, writing – original draft.

## Conflicts of Interest

The authors declare no conflicts of interest.

## Supporting information


**Table S1** Stirling Children’s Well‐being Scale (SCWBS).
**Table S2:** Warwick–Edinburgh Mental Well‐Being Scale (WEMWBS).
**Table S3:** Family Quality of Life Scale (FQOL).
**Table S4.** Child well‐being and family functioning during the early and late pandemic time periods.
**Table S5.** Stirling Children’s Well‐Being Scale (SCWBS) means with standard deviations (SD) for early and late pandemic time periods.
**Table S6.** Warwick–Edinburgh Mental Well‐Being Scale (WEMWBS) means with standard deviations (SD) for early and late pandemic time periods.
**Table S7.** Family quality of life (FQOL) mean scores with standard deviations (SD) for early and late pandemic time periods.
**Table S8.** Regression analysis evaluating the adjusted association between the pandemic time phases and Family Quality of Life Scale (FQOL) scores.

## Data Availability

Data will be shared, upon reasonable request, for academic purposes, with appropriate individuals who have obtained appropriate ethics permissions and data sharing agreements. Prior presentation of study data as poster: poster presented at the Pediatric Academic Societies conference on May 2–6, 2024, in Toronto, Canada
