# Supplementary material for: Child Well‐Being and Family Quality of Life During the COVID‐19 Pandemic
Source: Child Care Health Dev. 2025 Mar 14;51(2):e70063. doi: 10.1111/cch.70063 (PMC11908635; doi:10.1111/cch.70063)
Supplement: Supplementary file 1 — Table S1 Stirling Children’s Well‐being Scale (SCWBS). Table S2: Warwick–Edinburgh Mental Well‐Being Scale (WEMWBS). Table S3: Family Quality of Life Scale (FQOL). Table S4. Child well‐being and family functioning during the early and late pandemic time periods. Table S5. Stirling Children’s Well‐Being Scale (SCWBS) means with standard deviations (SD) for early and late pandemic time periods. Table S6. Warwick–Edinburgh Mental Well‐Being Scale (WEMWBS) means with standard deviations (SD) for early and late pandemic time periods. Table S7. Family quality of life (FQOL) mean scores with standard deviations (SD) for early and late pandemic time periods. Table S8. Regression analysis evaluating the adjusted association between the pandemic time phases and Family Quality of Life Scale (FQOL) scores. [file CCH-51-e70063-s001.docx]

**Supplemental Table 1: Stirling Children’s Well-being Scale (SCWBS)**

| **SCWBS item** | **Rating: 1 to 5** |
| --- | --- |
| I think good things will happen in my life | **1 - Never**  **2 - Not much of the time**  **3 - Some of the time**  **4 - Quite a lot of the time**  **5 - All of the time** |
| I've been able to make choices easily |  |
| I can find lots of fun things to do |  |
| I feel that I am good at some things |  |
| I think lots of people care about me |  |
| I think there are many things I can be proud of |  |
| I've been feeling calm |  |
| I've been in a good mood |  |
| I enjoy what each new day brings |  |
| I've been getting on well with people |  |
| I've been cheerful about things |  |
| I've been feeling relaxed |  |
| **Total score /60** |  |

**Supplemental Table 2: Warwick- Edinburgh Mental Well-Being Scale (WEMWBS)**

| **WEMWBS item** | **Rating: 1 to 5** |
| --- | --- |
| I've been feeling optimistic about the future | **1 - None of the time**  **2 - Rarely**  **3 - Some of the time**  **4 - Often**  **5 - All of the time** |
| I've been feeling useful |  |
| I've been feeling relaxed |  |
| I've been feeling interested in other people |  |
| I've had energy to spare |  |
| I've been dealing with problems well |  |
| I've been thinking clearly |  |
| I've been feeling good about myself |  |
| I've been feeling close to other people |  |
| I've been feeling confident |  |
| I've been able to make up my own mind about things |  |
| I've been feeling loved |  |
| I've been interested in new things |  |
| I've been feeling cheerful |  |
| **Total score /70** |  |

**Supplemental Table 3: Family Quality of Life Scale (FQOL)**

| **FQOL item** | **Rating: 1 to 5** |
| --- | --- |
| **Family Interaction** | **1 - Very Dissatisfied**  **2 - Dissatisfied**  **3 - Neither**  **4 - Satisfied**  **5 - Very Satisfied** |
| My family enjoys spending time together |  |
| My family members talk openly with each other |  |
| My family solves problems together |  |
| My family members support each other to accomplish goals |  |
| My family members show that they love and care for each other |  |
| My family is able to handle life's ups and downs |  |
| **Parenting** |  |
| My family members help the children learn to be independent |  |
| My family members help the children with schoolwork and activities |  |
| My family members teach the children how to get along with others |  |
| Adults in our family teach the children to make good decisions |  |
| Adults in my family know other people in the children's lives |  |
| Adults in my family have time to take care of the individual needs of every child |  |
| **Emotional Well-Being** |  |
| My family has the support we need to relieve stress |  |
| My family members have friends or others who provide support |  |
| My family members have some time to pursue our own interests |  |
| My family has outside help available to use to take care of special needs of all family members |  |
| **Physical/Material Well-Being** |  |
| My family gets medical care when needed |  |
| My family gets dental care when needed |  |
| My family members have transportation to get to the places they need to be |  |
| My family has a way to take care of our expenses |  |
| My family feels safe at home, work, school, and in our neighborhood |  |
| **Disability-Related Support** |  |
| My family member with a [mental health concern] has support to accomplish goals at school or at workplace |  |
| My family member with a [mental health concern] has support to accomplish goals at home |  |
| My family member with a [mental health concern] has support to make friends |  |
| My family has good relationships with the service providers who provide services and support to our family member with a [mental health concern] |  |
| **Total score /125** |  |

**Supplemental Table 4.** Child well-being and family functioning during the early and late pandemic time periods.

| **Outcome** | **Early pandemic**  **(March 15, 2020 – March 14, 2021)** | | **Late pandemic**  **(July 1, 2021 – June 30, 2022)** | | **Difference (95% CI)** | **Unadjusted**  ***P* value** |
| --- | --- | --- | --- | --- | --- | --- |
|  | **N** | **Mean (SD)** | **N** | **Mean (SD)** |  |  |
| **Well-Being** | | | | | | |
| Children < 13 years | 352 | 33.78 (8.04) | 317 | 33.35 (7.57) | -0.43  (-1.61, 0.76) | 0.48 |
| Children > 13 years | 297 | 36.68 (9.13) | 323 | 36.17 (7.99) | -0.53  (-1.87, 0.83) | 0.45 |
| **Family Quality of Life** | | | | | | |
| Total score | 628 | 96.93 (14.35) | 619 | 94.77 (14.99) | -2.16  (-3.79, -0.53) | 0.01 |
| Family interaction | 630 | 23.26 (4.41) | 621 | 22.71 (4.47) | -0.55  (-1.04, -0.06) | 0.03 |
| Parenting | 630 | 23.92 (3.87) | 621 | 23.32 (3.89) | -0.60  (-1.03, -0.17) | 0.007 |
| Emotional well-being | 629 | 13.89 (3.41) | 621 | 13.58 (3.54) | -0.31  (-0.69, 0.08) | 0.12 |
| Physical and material  well-being | 629 | 21.42 (3.08) | 621 | 20.85 (3.31) | -0.56  (-0.92, -0.21) | 0.002 |
| Disability-related support | 630 | 14.45 (3.18) | 619 | 14.30 (3.11) | -0.15  (-0.50, 0.20) | 0.39 |

**Supplemental Table 5.** Stirling Children's Well-Being Scale (SCWBS) means with standard deviations (SD) for early and late pandemic time periods**.**

| **SCWBS item** | **Early pandemic**  **(n=352)** | **Late pandemic**  **(n=317)** | **Overall**  **(n=669)** | **Mean difference (95% CI)** |
| --- | --- | --- | --- | --- |
| I think good things will happen in my life | 2.83 (0.94) | 2.82 (0.90) | 2.82 (0.92) | -0.01 (-0.15, 0.13) |
| I've been able to make choices easily | 2.82 (0.95) | 2.76 (0.94) | 2.79 (0.95) | -0.06 (-0.20, 0.08) |
| I can find lots of fun things to do | 2.96 (0.98) | 2.91 (0.90) | 2.94 (0.95) | -0.04 (-0.19, 0.10) |
| I feel that I am good at some things | 3.02 (1.02) | 3.00 (1.02) | 3.01 (1.02) | -0.01 (-0.17, 0.14) |
| I think lots of people care about me | 3.18 (1.13) | 3.08 (1.08) | 3.13 (1.11) | -0.10 (-0.27, 0.07) |
| I think there are many things I can be proud of | 2.87 (0.98) | 2.85 (1.01) | 2.86 (0.99) | -0.02 (-0.17, 0.13) |
| I've been feeling calm | 2.51 (0.87) | 2.44 (0.86) | 2.48 (0.86) | -0.07 (-0.20, 0.06) |
| I've been in a good mood | 2.66 (0.87) | 2.64 (0.80) | 2.65 (0.84) | -0.01 (-0.14, 0.11) |
| I enjoy what each new day brings | 2.72 (0.94) | 2.66 (0.87) | 2.69 (0.9) | -0.06 (-0.2, 0.08) |
| I've been getting on well with people | 3.03 (0.91) | 3.00 (0.85) | 3.01 (0.88) | -0.03 (-0.16, 0.11) |
| I've been cheerful about things | 2.75 (0.85) | 2.73 (0.82) | 3.01 (0.88) | -0.02 (-0.15, 0.10) |
| I've been feeling relaxed | 2.45 (0.86) | 2.45 (0.84) | 2.45 (0.85) | 0.01 (-0.12, 0.14) |
| **Total score, mean (SD)** | 33.78 (8.04) | 33.35 (7.57) | 33.58 (7.82) | -0.43 (-1.61, 0.76) |

**Supplemental Table 6.** Warwick-Edinburgh Mental Well-Being Scale (WEMWBS) means with standard deviations (SD) for early and late pandemic time periods.

| **WEMWBS item** | **Early pandemic**  **(n=297)** | **Late pandemic**  **(n=323)** | **Overall**  **(n=620)** | **Mean difference**  **(95% CI)** |
| --- | --- | --- | --- | --- |
| I've been feeling optimistic about the future | 2.61 (0.91) | 2.57 (0.85) | 2.59 (0.88) | -0.05 (-0.18, 0.09) |
| I've been feeling useful | 2.45 (0.88) | 2.50 (0.87) | 2.48 (0.88) | 0.04 (-0.09, 0.18) |
| I've been feeling relaxed | 2.33 (0.87) | 2.44 (0.81) | 2.38 (0.84) | 0.11 (-0.02, 0.24) |
| I've been feeling interested in other people | 2.91 (1.04) | 2.77 (1.00) | 2.84 (1.02) | -0.14 (-0.30, 0.03) |
| I've had energy to spare | 2.31 (0.94) | 2.34 (0.95) | 2.33 (0.95) | 0.02 (-0.13, 0.17) |
| I've been dealing with problems well | 2.52 (0.92) | 2.35 (0.85) | 2.43 (0.89) | -0.17 (-0.31, -0.03) |
| I've been thinking clearly | 2.69 (0.91) | 2.62 (0.82) | 2.65 (0.86) | -0.07 (-0.20, 0.07) |
| I've been feeling good about myself | 2.38 (0.97) | 2.32 (0.85) | 2.35 (0.91) | -0.06 (-0.20, 0.09) |
| I've been feeling close to other people | 2.81 (0.89) | 2.77 (0.87) | 2.79 (0.88) | -0.03 (-0.17, 0.10) |
| I've been feeling confident | 2.38 (0.98) | 2.32 (0.84) | 2.35 (0.91) | -0.06 (-0.20, 0.09) |
| I've been able to make up my own mind about things | 3.04 (1.01) | 2.95 (0.90) | 2.99 (0.95) | -0.08 (-0.23, 0.07) |
| I've been feeling loved | 3.27 (0.96) | 3.29 (0.89) | 3.28 (0.92) | 0.02 (-0.13, 0.16) |
| I've been interested in new things | 2.54 (1.04) | 2.50 (1.00) | 2.52 (1.02) | -0.04 (-0.20, 0.12) |
| I've been feeling cheerful | 2.46 (0.87) | 2.42 (0.85) | 2.44 (0.86) | -0.04 (-0.17, 0.10) |
| **Total score, mean (SD)** | 36.68 (9.13) | 36.16 (7.99) | 36.41 (8.55) | -0.52 (-1.87, 0.83) |

**Supplemental Table 7.** Family quality of life (FQOL) mean scores with standard deviations (SD) for early and late pandemic time periods.

| **FQOL item** | **Early pandemic**  **(n=628)** | **Late pandemic**  **(n=619)** | **Overall**  **(n=1247)** | **Mean difference (95% CI)** |
| --- | --- | --- | --- | --- |
| **Family Interaction** | 23.26 (4.41) | 22.71 (4.47) | 22.98 (4.45) | -0.55 (-1.04, -0.06) |
| My family enjoys spending time together | 3.89 (0.97) | 3.80 (0.98) | 3.85 (0.98) | -0.09 (-0.20, 0.02) |
| My family members talk openly with each other | 3.88 (0.99) | 3.77 (1.00) | 3.83 (1) | -0.11 (-0.22, 0) |
| My family solves problems together | 3.68 (0.99) | 3.59 (1.00) | 3.64 (1) | -0.09 (-0.20, 0.02) |
| My family members support each other to accomplish goals | 3.94 (0.92) | 3.85 (0.91) | 3.90 (0.91) | -0.08 (-0.19, 0.02) |
| My family members show that they love and care for each other | 4.16 (0.87) | 4.11 (0.88) | 4.14 (0.88) | -0.05 (-0.15, 0.05) |
| My family is able to handle life's ups and downs | 3.70 (0.89) | 3.57 (0.95) | 3.63 (0.92) | -0.13 (-0.23, -0.03) |
| **Parenting** | 23.92 (3.87) | 23.32 (3.89) | 23.62 (3.89) | -0.60 (-1.03, -0.17) |
| My family members help the children learn to be independent | 4.00 (0.83) | 3.91 (0.83) | 3.96 (0.83) | -0.09 (-0.18, 0) |
| My family members help the children with schoolwork and activities | 3.82 (0.99) | 3.67 (1.04) | 3.75 (1.02) | -0.16 (-0.27, -0.05) |
| My family members teach the children how to get along with others | 4.08 (0.83) | 3.98 (0.85) | 4.03 (0.84) | -0.10 (-0.20, -0.01) |
| Adults in our family teach the children to make good decisions | 4.12 (0.81) | 4.06 (0.78) | 4.09 (0.79) | -0.06 (-0.15, 0.03) |
| Adults in my family know other people in the children's lives | 4.08 (0.82) | 3.97 (0.81) | 4.02 (0.82) | -0.11 (-0.20, -0.02) |
| Adults in my family have time to take care of the individual needs of every child | 3.81 (0.95) | 3.74 (0.96) | 3.78 (0.96) | -0.08 (-0.18, 0.03) |
| **Emotional Well-Being** | 13.89 (3.41) | 13.58 (3.54) | 13.74 (3.48) | -0.31 (-0.69, 0.08) |
| My family has the support we need to relieve stress | 3.25 (1.11) | 3.12 (1.13) | 3.18 (1.12) | -0.13 (-0.26, -0.01) |
| My family members have friends or others who provide support | 3.59 (1.03) | 3.64 (1.03) | 3.62 (1.03) | 0.05 (-0.07, 0.16) |
| My family members have some time to pursue our own interests | 3.67 (1.05) | 3.57 (1.06) | 3.62 (1.05) | -0.09 (-0.21, 0.02) |
| My family has outside help available to use to take care of special needs of all family members | 3.38 (1.16) | 3.25 (1.18) | 3.31 (1.17) | -0.13 (-0.26, 0) |
| **Physical/Material Well-Being** | 21.42 (3.08) | 20.85 (3.31) | 21.14 (3.21) | -0.56 (-0.92, -0.21) |
| My family gets medical care when needed | 4.47 (0.76) | 4.34 (0.89) | 4.41 (0.83) | -0.13 (-0.22, -0.04) |
| My family gets dental care when needed | 4.37 (0.81) | 4.22 (0.91) | 4.29 (0.86) | -0.15 (-0.25, -0.06) |
| My family members have transportation to get to the places they need to be | 4.04 (0.94) | 3.90 (1.03) | 3.97 (0.99) | -0.14 (-0.25, -0.03) |
| My family has a way to take care of our expenses | 4.33 (0.93) | 4.29 (0.94) | 4.31 (0.94) | -0.05 (-0.15, 0.06) |
| My family feels safe at home, work, school, and in our neighborhood | 4.20 (0.88) | 4.11 (0.88) | 4.15 (0.88) | -0.09 (-0.19, 0.01) |
| **Disability-Related Support** | 14.45 (3.18) | 14.3 (3.11) | 14.38 (3.14) | -0.15 (-0.50, 0.20) |
| My family member with a [mental health concern] has support to accomplish goals at school or at workplace | 3.52 (1.01) | 3.46 (0.98) | 3.49 (0.99) | -0.06 (-0.17, 0.05) |
| My family member with a [mental health concern] has support to accomplish goals at home | 3.66 (0.94) | 3.63 (0.94) | 3.64 (0.94) | -0.03 (-0.13, 0.08) |
| My family member with a [mental health concern] has support to make friends | 3.52 (1.00) | 3.53 (0.96) | 3.53 (0.98) | 0.01 (-0.1, 0.11) |
| My family has good relationships with the service providers who provide services and support to our family member with a [mental health concern] | 3.75 (0.91) | 3.69 (0.91) | 3.72 (0.91) | -0.06 (-0.16, 0.04) |
| **Total score, mean (SD)** | 96.93 (14.35) | 94.77 (14.99) | 95.86 (14.7) | -2.16 (-3.79, -0.53) |

**Supplemental Table 8.** Regression analysis evaluating the adjusted association between the pandemic time phases and Family Quality of Life Scale (FQOL) scores.

|  | **Adjusted mean difference (95% CI)** | ***P* value** |
| --- | --- | --- |
| **History of developmental delay** | | |
| Yes | -4.01 (-6.43, -1.60) | 0.001 |
| No | Reference |  |
| **Currently receiving outpatient mental health care** | | |
| Yes | 2.29 (0.1, 4.48) | 0.04 |
| No | Reference |  |
| **Prior hospitalization for mental health** | | |
| Yes | -5.41 (-7.69, -3.13) | <0.001 |
| No | Reference |  |
| **Discharge diagnosis** | | |
|  |  |  |
| Mood (affective disorders) | 3.60 (1.67, 5.52) | <0.001 |
| No diagnosis of Mood (affective disorders) | Reference |  |
| Neurotic, stress-related, and somatoform disorders | 2.61 (0.73, 4.49) | 0.006 |
| No diagnosis of Neurotic, stress-related, and somatoform disorders | Reference |  |
| **Pandemic time-period** | | |
| Late | -2.27 (-3.90, -0.64) | 0.006 |
| Early | Reference |  |

ED, Emergency Department.
